# Cultural influence of social information use in pedestrian road-crossing behaviours

**DOI:** 10.1098/rsos.160739

**Published:** 2017-02-15

**Authors:** Marie Pelé, Caroline Bellut, Elise Debergue, Charlotte Gauvin, Anne Jeanneret, Thibault Leclere, Lucie Nicolas, Florence Pontier, Diorne Zausa, Cédric Sueur

**Affiliations:** 1 Ethobiosciences, Research and Consultancy Agency in Animal Wellbeing and Behaviour, Strasbourg, France; 2 Département Ecologie, Physiologie et Ethologie, Centre National de la Recherche Scientifique, Strasbourg, France; 3 Institut Pluridisciplinaire Hubert Curien, Université de Strasbourg, Strasbourg, France; 4 Primate Research Institute, Kyoto University, Inuyama, Japan

**Keywords:** culture, traffic injury, risk, decision-making, collective behaviour, tradition

## Abstract

Social information use is common in a wide range of group-living animals, notably in humans. The role it plays in decision-making could be a key to understanding how social groups make collective decisions. The observation of road-crossing behaviours in the presence of other individuals is an ideal means to study the influence of social information on decision-making. This study investigated the influence of culture on social information used by pedestrians in a potentially dangerous scenario, namely road crossing. We scored the collective crossing of pedestrians at four locations in Nagoya (Japan) and three locations in Strasbourg (France). French pedestrians cross against the lights much more often (41.9%) than Japanese ones (2.1%). Individuals deciding to cross the road were strongly influenced by the behaviour and the presence of other pedestrians, especially in Japan, where a stronger conformism was noted. However, Japanese pedestrians were half as likely to be influenced by social information as their French counterparts when crossing at the red light, as they were more respectful of rules. Men show riskier behaviour than women (40.6% versus 25.7% of rule-breaking, respectively), deciding quickly and setting off earlier than women. Further related studies could help target specific preventive, culture-specific solutions for pedestrian safety.

## 1. Introduction

The acquiring of information by animals, including human beings, should result in a decision that is as optimal as possible and ensure that the fitness of individuals either increases or remains stable. When making a decision, an individual has three major constraints: (i) the time it has to decide, (ii) the information concerning each alternative (on which the decision accuracy depends) and (iii) the risk involved in each alternative [1,2].

The information an individual obtains from its environment can be social or non-social (i.e. gained from personal observations). Social information can be produced advertently (a signal) or inadvertently (a cue), and its use might allow individuals to adapt to their environment faster and/or better than when collecting personal information alone [3]. However, the use of social information has also been proved to be disadvantageous in some animal species [4].

Many studies have shown that humans are able to respond to social information by observing the behaviours of their conspecifics. Communicating human groups perform better during social foraging experiments than non-communicating groups [5]. Individuals may use inadvertent social information such as age [6,7] or speed [8] to identify the more performant individuals and follow them. This social information transmission can lead to contagious and collective behaviours such as those observed in human crowds and pedestrian traffic [9,10].

Walking in the street is a daily necessity for the majority of humans. Although walking can be considered a safe behaviour in the light of the number of times people cross the road without getting struck by a vehicle, the circumstances, environment and types of road-use behaviours (both pedestrians and drivers) can make it a high-risk behaviour. This activity causes the largest number of pedestrian accidents and causes the most severe injuries [11]. King *et al.* [12] showed that crossing against the red light in Australia entails a collision risk that is eight times higher than crossing at the green light (another study showed that it is 20 times higher [13]). Of the Mumbai (India) citizens who died in road accidents between 2008 and 2012, 57% were pedestrians, a figure so high that it was the subject of an article in the *Mumbai Mirror* entitled *Pedestrian is the most endangered species in Mumbai* [14]. Pedestrian behaviour has become an important research area as the size of human population living in big cities increases, and their safety is considered to be a priority in infrastructure improvements projects. The faster a pedestrian decides to cross, the riskier the decision will be, as the pedestrian takes less time to obtain information before stepping off the kerb. However, this decision might be influenced by different social and non-social factors [15]. If some pedestrians are crossing against the red light, this unreliable source of information might lead others to risk injury [16]. This probability to follow another pedestrian across the road depends on several factors, such as the spatial and social proximity to this pedestrian or the gender of pedestrians [8]. In some cases, individuals following unreliable social information may start to cross and then return to the pavement after evaluating the danger on their own criteria and deciding that crossing is too risky [8]. Here, the incorrect decisions to cross are based purely on social information use. Information cascades or amplification processes may result in several pedestrians crossing against the red light without evaluating the risk. An information cascade occurs when a pedestrian observes the actions of others and then, despite possible contradictions in his/her personal information, does likewise, i.e. crosses the road. The amplification process is quite similar, except that in this case the probability to cross increases with the number of individuals crossing. Therefore, even if their decision is wrong, the more individuals are already crossing, the more likely it is that the remaining individuals will follow them. This study attempts to identify the mechanisms underlying collective road-crossing behaviours and investigates how these mechanisms and the use of social information are affected by different factors such as the number of pedestrians, their time of arrival at the crossing point, their gender, age and culture.

Existing literature claims that men often decide more quickly and take more risks than women [16,17]. Women appear to focus more on the behaviour of other pedestrians (i.e. on social information), while men give priority to observing the road and the cars (personal information) [18]. Young and old people seem to break pedestrian rules more often and show more risky behaviour than middle-aged people [19,20], but this depends on the situation (alone at the kerb, whether crossing points are signalled or not, etc. [17]). Risky behaviours in senior citizens seem to vary according to the decline of their perception and mobility. Although children may also be less able to evaluate risk, young male adults are highly likely to break the rules for crossing the road due to a willingness to participate in risky and competitive interactions [21].

However, we have very little data concerning how the national traditions of individuals could influence their perception of risks when crossing the road [22]. Culture is defined as the way of life, and how ‘knowledge, belief, art, morals, law, custom and any other capabilities and habits' are acquired by man as a member of society [23]. It is therefore possible that our culture might influence us in our way of crossing the road to the same extent as our gender or our age. Ultra-orthodox pedestrians seem to more often break the rules than secular pedestrians do [16]. Another report has also described the significant effect of culture on rule-breaking by pedestrians in Singapore and Beijing, two cities in Asian countries [24]. Strong differences were observed between French and Japanese citizens in our previous study of cultural differences for single pedestrians (i.e. excluding couples or groups) crossing at safe or unsafe sites [17]. Here we studied the collective road-crossing behaviours of pedestrians between these same two different countries with very different cultures. The Western culture of countries like France is considered to involve more risky behaviours, less tolerance for hierarchical relationships or for following rules, and more individualism than in Asian cultures [25,26]. Individualistic culture tends to make people more autonomous, while collectivist societies seem to drive individuals to be more cautious [27]. These differences in so-called social conventions between different countries might emerge from institutional mechanisms but could also spontaneously emerge from self-organized and local interactions. If the latter is true, choosing one alternative is not better than choosing the other [28], such as driving on the left or the right. However, we have no idea how these different cultures might influence social information use, especially in the case of road crossing, a high-risk behaviour when the red light is ignored.

We studied four sites in the city of Nagoya (Japan) and three sites in the city of Strasbourg (France) which were all comparable in terms of traffic speed and number of pedestrians. We first analysed the rule-breaking rate and then the time at which pedestrians started crossing according to different variables:

(1) Environmental factors such as the light colour, the number of lanes, the proximity to the kerb or the time elapsed since the last car passed. We expected the number of lanes to negatively affect the probability of crossing against the lights. We also expected pedestrians located closer to the kerb to cross more quickly. Fewer individuals were expected to cross against the red light, and this phenomenon was expected to vary according to the country.
(2) Personal variables such as age and gender, but also the time taken to arrive at the road crossing, were expected to affect the arrival order and the waiting time. As described in previous studies, we expected an effect of age and gender on the probability to break the rules and to follow social information. We also expected pedestrians who had waited longer at the red light to start crossing earlier than those who had waited less [29].
(3) Social variables, namely the country (culture), the number of pedestrians waiting at the kerb, whether or not pedestrians are accompanied and the behaviours of other pedestrians (i.e. crossing at the red or green light). We suggest that the country should have an impact on crossing behaviours, with Japanese pedestrians showing more care for other individuals (social information), whereas French people are expected to be more individualistic (personal information). Following information cascades and amplification behaviours [9,30], we also expected more pedestrians to start crossing as the number of individuals already crossing the road increased [31]. Finally, the probability that an individual will follow a pedestrian who is crossing against the red light should depend on the country, with Japanese pedestrians showing less risky behaviour and/or following the rules more than their French counterparts.

## 2. Methods

### 2.1. Study sites

We observed pedestrian behaviours at three sites in Strasbourg, France and at four sites in Nagoya, Japan. Details about each site are given in table 1. Pictures for each site are available in the electronic supplementary material file and in Google View®. These sites all permitted the observation of collective road crossings involving at least 10 pedestrians at a time. The speed of vehicles on each site was limited to 50 km h^−1^. There was no difference in pedestrian crossing speed between the sites (permutation test for independent samples: max*T* = 2.22, *p* = 0.168). At some sites, vehicles were allowed to turn left or right despite the green light for pedestrians, but the drivers were aware that crossing pedestrians had priority. Moreover, turning vehicles travel much slower than vehicles that are heading straight on. However, the driver of an approaching vehicle may be less careful if pedestrians cross at the red light, as he/she has the right to pass. The risk to pedestrians is therefore much higher when crossing at the red light. There was no button for pedestrians to trigger the green light at any of the sites studied.

**Table 1.** Information about the studied sites in France and in Japan. Road-crossing speed was estimated by scoring the crossing speed of 20 random pedestrians for each site.

	France-Strasbourg			Japan-Nagoya			
sites	train station	Pont des Corbeaux	Place Broglie	train Station	Maruei	Excelco	Osu-Kannon
coordinates	48.584474, 7.736135	48.579509, 7.750745	48.584559, 7.748628	35.170824, 136.884328	35.168638, 136.905740	35.166891, 136.907284	35.159316, 136.901697
lanes	2 × 1	2 × 2	2 × 1	2 × 3	1 × 1	2 × 1	2 × 1
turning vehicles	no	yes	no	yes	yes	no	no
mean pedestrian flow per hour	667	612	850	480	645	869	814
mean road-crossing speed (m s^−1^)	0.96 ± 0.05	1.11 ± 0.29	1.01 ± 0.16	1.10 ± 0.22	1.15 ± 0.21	0.98 ± 0.21	1.07 ± 0.18
dates of scoring	2 July–7 July 2014	1 October–25 October 2014	15 February–9 March 2015	13 June–5 July 2011		27 January–5 February 2015	

### 2.2. Data scoring

Data were scored over a 6 day period for each site, for 1 h per day during working days, hours and weeks to ensure that data excluded movements generated by tourism, festivals, etc. This scoring duration is sufficiently ample to provide a large dataset [8,17,31]. Video cameras were set up in order to score the light colour and were placed in locations ensuring that crossing pedestrians were visible at all times. Behavioural sampling was used to score the crossing of pedestrians in one direction only, i.e. that recorded by the camera. Pedestrians were not informed about the purpose of the study. As both cities are touristic, pedestrians are accustomed to seeing tourists taking pictures or videos. We did not observe any difference in the way pedestrians behaved when they saw the camera. We purposely did not take any other equipment such as counters or pocket PC in order to avoid influencing pedestrian behaviour. When observation of road-crossing behaviour was hampered by a visual obstacle (i.e. a car or a truck in front of the video camera), we removed this behaviour and the behaviours occurring immediately before and after it from the analyses. We also removed data in which cyclists were among the pedestrians or where tourists were present. Tourists were easily differentiated from citizens, as they were in large groups accompanied by a guide, were dressed differently from citizens and carried specific equipment (guidebook, map, camera, etc.).

### 2.3. Research ethics

Our methodological approach solely involved anonymous observations and anonymous data scores. Our protocol followed the ethical guidelines of our institutions (IPHC, Strasbourg, France and PRI, Kyoto University, Japan) and we received ethical approval from these institutions to carry out our study. All data were anonymous, and individuals were given sequential numerical identities according to the time of the road crossing and the arrival/departure order of crossing. Pedestrians had the possibility to be informed about the study by an information medium in their language (Japanese or French). They were also provided with an email address and phone number to contact our institution at a later date if desired. Persons who refused to participate in the study were removed from the data (i.e. we deleted the crossing concerned).

### 2.4. Data analysing

Data are available from Dryad repository (http://dx.doi.org/10.5061/dryad.tc365). Two kinds of data were analysed on the videos: (i) crossing against the red light and (ii) collective road crossing at the red or green light.

Of course, collective road-crossing can involve crossing at the red light—the two behaviours are not exclusive. In the first case, i.e. crossing at the red light, we wanted to analyse the individual rule-breaking behaviour independently of collective behaviours, whereas in the second part, i.e. ‘collective road-crossing’, we aimed to analyse the collective processes and identify those influencing an individual's decision to follow others across the road at a green or red light.

(1) Crossing against the red light: for each site, two 10 min periods were chosen at random for each scored day in order to score each crossing behaviour. This led to the analysis of 5445 road crossings, of which 3814 were in France and 1631 in Japan. For each pedestrian crossing the road, we scored the site, country, number of lanes, light colour, and gender and age of the pedestrian. We also recorded whether the pedestrian was accompanied by one person or more. The age of pedestrians was estimated at 10 year intervals, from 0–9, 10–19 […] to 70–89. A pedestrian is considered to be accompanied when he/she is talking with another person or walking very closely and synchronously with her/him, arriving at the road and crossing it at the same time.
(2) Collective road crossing at red or green light: all 6 h of data were analysed for each site. We scored the behaviours of pedestrians during collective crossings, which were defined as those involving two or more pedestrians at the green light (occurring without prior jaywalking by pedestrians) or at the red light, when the time between the departures of the two pedestrians was lower than the mean road-crossing time indicated in table 1 for each site. Any collective road crossings in which pedestrians crossing at the red light were immediately followed by pedestrians crossing at the next green light were discarded from data: in these specific cases, it is impossible to confirm whether pedestrians crossing at the green light were influenced by the pedestrians crossing against the red light. We then separated collective crossings at the green light from collective crossings at the red light. We scored collective road crossings for 3666 pedestrians, of which 2082 crossed at the green light. The remaining 1584 pedestrians either crossed at the red light or crossed at the green light but were preceded by pedestrians crossing at the red light. After discarding the latter cases and cleaning the data, 963 cases of illegal road-crossing behaviours remained.

We decided to only analyse the crossing behaviours (at the green or at the red light) of the first 10 pedestrians for two reasons: first, it was very difficult to analyse the time of crossing and order of crossing when there were more than 10 pedestrians crossing the road collectively, and second, the processes influencing the road-crossing behaviours of the first 10 pedestrians are different from those influencing the next 10 or 20 pedestrians, with a higher influence of individual variables in the first 10 pedestrians compared with a more global amplification process for subsequent individuals crossing the road [30,32]. Similarly, we analysed only data for pedestrians who were present at the crossing when the light colour changes, or who either stopped or decreased their walking speed as they approached the crossing, as we wanted to assess how these specific factors (i.e. light colour, waiting time, number of pedestrians) influenced their decisions to cross the road.

**Figure 1.**
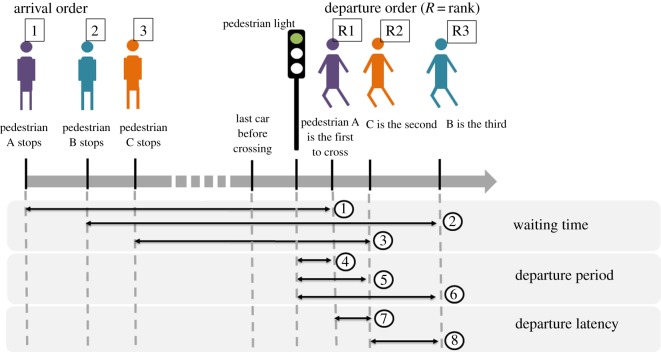
Visual explanation of the different variables we scored. ❶, ❷ and ❸ are the waiting times of pedestrians (A–C), respectively. ❹, ❺ and ❻ are the departure periods of pedestrians (A–C), respectively. ❼ and ❽ are the departure latencies of C (after the departure of A) and B (after the departure of C), respectively. As illustrated in this figure, arrival order can differ from departure order.

All indicated times are in hundredths of a second. For each pedestrian crossing collectively, we scored the following variables (see figure 1 for a visual explanation of the different scored variables):

— The departure period, i.e. the period between the previous light colour change and the moment the pedestrian starts crossing the road. This variable is only considered for pedestrians crossing at the green light.
— The departure latency, i.e. the time elapsed between the departure of pedestrian *p* and previous pedestrian *p* − 1*.* This variable is therefore not applicable for the first pedestrian to leave, whatever the colour of the light.
— The departure order of pedestrians, where the first pedestrian to leave is ranked as 1, the second as 2, and so on.
— The gender (male or female).
— The age, estimated at 10-year intervals from 0–9, 10–19 […] to 70–89.
— The country (France or Japan).
— The number of lanes.
— The light colour when crossing (red or green).
— The closeness to the kerb (evaluated as lines of pedestrians at increasing distances from the kerb; line 1 being the closest line to the kerb).
— The total number of pedestrians involved in the road-crossing event.
— The waiting time, i.e. the time between the moment a pedestrian stops at the light and the moment he/she starts crossing the road.
— The arrival order, i.e. the order in which pedestrians arrive at the light before crossing, where the first pedestrian to arrive is ranked as 1, the second as 2 and so on.
— The last car time, i.e. the time elapsed between the passage of the last car on the pelican crossings and the moment when each pedestrian starts to cross.

### 2.5. Hierarchical cluster analysis

We performed a hierarchical cluster analysis following a principal component analysis in order to explore the distribution of variables and data. The hierarchical cluster analysis (automatic selection) showed that the data are clustered in five clusters and this distribution mainly depends on the arrival order, the waiting time, the closeness to the kerb and the total number of pedestrians. Figures and tables about contributions of each variable as well as statistical values are given in the supplementary information file (electronic supplementary material, figure S8 and tables S2–S5).

### 2.6. Statistical analyses

A generalized linear model (GzLM) was used to test the influence of the independent variables on our five following response variables. We first analysed road crossing with the dependent variable, i.e. the light colour (red or green), using a binomial law. In a second step, the collective road-crossing behaviours at the green light were analysed by testing the order of departure (Poisson law), the departure period of the first pedestrian to cross (Gaussian law), and the departure period and the departure latency of other pedestrians (Gaussian law). To assess whether pedestrians are influenced in their way to cross by other pedestrians crossing at the red or at the green light, we also tested the departure latency of pedestrians in combined datasets for pedestrians crossing at the red and at the green light.

For each GzLM, we ran multi-model inferences to compare and rank candidate models according to (i) their respective Akaike information criterion after correction for small sample sizes (AICc) and (ii) normalized Akaike weights (AICw) [33]. ΔAICc is the difference in AICc between one given model and the model with the lowest AIC. The AIC weight indicates the probability of a given model being the best among candidate models. Models with a ΔAICc < 4 were considered equally possible candidates and then their statistics averaged. The null model was included as a possible candidate but was never among the models with lowest AICc. Averaged model coefficients were obtained for models with a ΔAICc < 4. Model inference and averaging were carried out with the R package ‘MuMIn’ [34]. We checked for multicollinearity of the predictor variables by calculating the variance inflation factor (vif). In all cases, the predictor variables had a vif value close to 1.00, indicating that the predictor variables were not correlated. The highest vif values were observed for arrival order (2.21) and waiting time (2.51), but these values remain far from the accepted threshold of 4, beyond which variables cannot be tested. We also tested models with interactions to check how some factors influence the results and impacts of others. We used a Wald test to check whether these models with interactions were the most suitable to explain variance of data. The significance level was set at 0.05. Statistical analyses were performed in R 2.15 [35].

## 3. Results

The relative importance of each variable (rvi) is given in table 2. The values indicate the number of times a variable was present in the best models (ΔAICc < 4). A summary of the GzLM with *z* and *p*-values is presented in the supplementary information section (electronic supplementary material, table S1).

**Table 2.** Relative importance of each independent variable (rvi) in the different models, and the sum of Akaike weights over all models including the explanatory variable. This table gives the extent to which each variable plays a role by explaining variance in the best models (ΔAICc < 4). An rvi value equalling 1 indicates that the variable is present in all best models and plays a major role in explaining the variance of dependent variables. Grey cells indicate that the independent variable was not tested in the corresponding model.

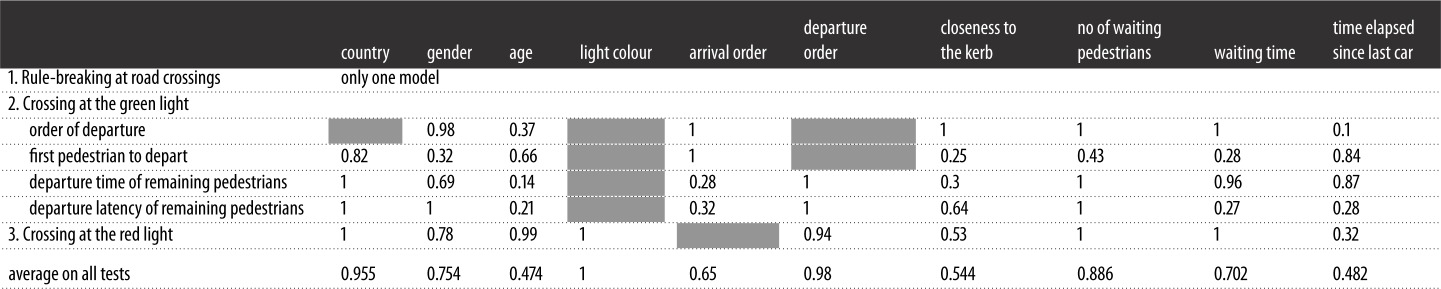

### 3.1. Crossing against the red light at road crossings

**Figure 2.**
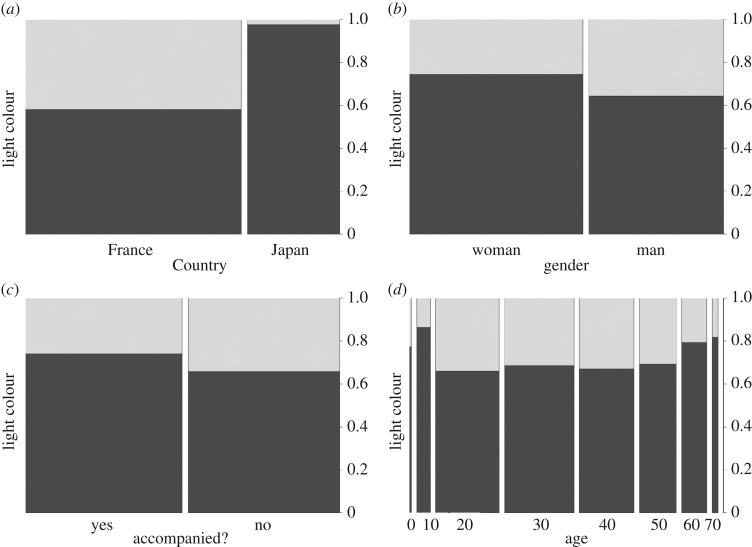
(*a*--*d*) Rate of crossing at the green light (dark grey) or at the red light (light grey) according to different factors. The width of each bar indicates the number of data items.

Among 5445 road crossings analysed (3814 in France, 1631 in Japan), we observed 1636 crossings at the red light and 3809 at the green light, meaning that 30% of crossings were illegal. However, this rate of rule-breaking is different for France (41.9%) and Japan (only 2.1%), (estimate = −4.08, *z*-value = −23.6, *p* < 0.0001, figure 2*a*). It increases to 76.3% in France but only 5.7% in Japan when only pedestrians arriving at the kerb at the red light are considered (we removed data for pedestrians arriving when the light was green, as they could not cross against the light). The low number of transgressions in Japan is reflected by the lack of effect for interactions between the country and age and/or gender. However, when Japanese and French data are combined, the rate of rule-breaking is affected by gender (male: estimate = 0.31, *z*-value = −4.42, *p* < 0.0001, figure 2*b*), accompanying pedestrians (single: estimate = 0.30, *z*-value = −4.07, *p* < 0.0001, figure 2*c*) and the number of lanes (estimate = −0.54, *z*-value = −14.67, *p* < 0.0001). Men are more prone to cross at the red light (40.6% of rule-breaking) than women (25.7%). More single individuals crossed against the red light (34.3% of rule-breaking) than accompanied pedestrians (30%). Finally, the above statistics reveal that the higher the number of lanes, the less likely it became that pedestrians would cross at the red light. Age has also an effect on the probability to cross against the red light, with a specifically higher incidence for individuals between 20 and 30 years old (estimate > |0.59|, *z*-value > |1.54|, *p* < 0.014, figure 2*d*). Other age classes are not statistically different from each other (estimate = −0.71, *z*-value = −1.7, *p* = 0.12), with the exception of individuals under the age of 10, who show a tendency for the lowest number of crossings against the red light (estimate = −0.71, *z*-value = −1.7, *p* = 0.088).

### 3.2. Crossing collectively at the green light

#### 3.2.1. Order of departure

**Figure 3.**
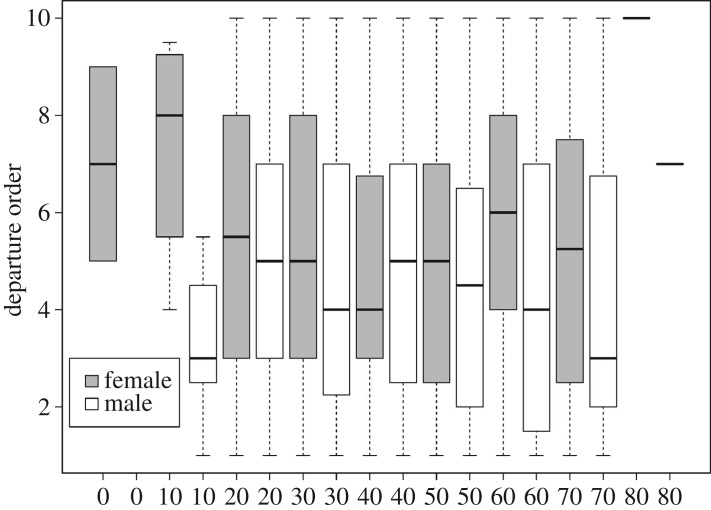
Departure order according to age (from [0, 10] to [80, 90]) and gender. Men tend to depart first more often than women, but age does not influence the departure order of individuals. On the *x* axis, age classes appear twice, one for females and one for males even if there are no data in one class.

The order of departure when the light turns green is influenced by the closeness to the kerb (estimate = 0.15, *z*-value = 12.00, *p* < 0.0001), then the waiting time (estimate = 2.99 × 10^−5^, *z*-value = 6.58, *p* < 0.0001) and the arrival order (estimate = 0.02, *z*-value = 5.66, *p* < 0.0001). It means that the sooner pedestrians arrive at the crossing point and the longer they wait, the more probable it is that they will be the first to start crossing. Men also started crossing sooner than women (estimate = −0.07, *z*-value = −2.9, *p* = 0.0003, figure 3). Age did not influence the order of departure (age categories compared with age [0, 10]: estimate < |0.42|, *z*-value < |1.35|, *p* > 0.175, figure 2), meaning that children or senior citizens are not among the first or last to start crossing. Adding the country variable as an interaction with gender and age did not explain variance any better than the model without any interactions (Wald test: d.f. = 3, *F* = 1.15, *p* = 0.327).

#### 3.2.2. First pedestrian to depart

The first pedestrian to leave was the one who arrived first at the kerb (estimate = 7.22 × 10^−4^, *z*-value = 3.2, *p* = 0.001), and the one who waited for a long period of time between the last car and the light change (estimate = −6.14 × 10^−8^, *z*-value = 2.27, *p* = 0.024). French citizens were faster to depart than their Japanese counterparts (estimate = −2.63 × 10^−3^, *z*-value = −2.25, *p* = 0.024). The moment the first pedestrians chose to leave was not influenced by the number of waiting pedestrians (estimate = 1.31 × 10^−4^, *z*-value = −1.23, *p* = 0.22), gender (male: estimate = 8.45 × 10^−4^, *z*-value = 0.858, *p* = 0.39), the closeness to the kerb (estimate = −3.13 × 10^−4^, *z*-value = 0.33, *p* = 0.74) or by the waiting time (estimate = −6 × 10^−4^, *z*-value = 0.47, *p* = 0.63). Nor did age influence the moment the first pedestrian chose to leave (estimate < |0.7|, *z*-value < 0.9, *p* > 0.36). There was no effect of interaction between the country and other factors, and the model with interactions did not explain variance any better than that without interactions (Wald test: d.f. = 2, *F* = 2.17, *p* = 0.116).

#### 3.2.3. Departure of remaining pedestrians

The departure period of remaining pedestrians was, of course, mainly influenced by the number of waiting pedestrians (estimate = 1.21 × 10^−4^, *z*-value = 9.6, *p* < 0.0001), then the waiting time (estimate = −6.15 × 10^−8^, *z*-value = 2.64, *p* = 0.008), by the time elapsed since the last car passed the crossing (estimate = −6.27 × 10^−9^, *z*-value = 2.23, *p* = 0.02) and finally by the country (Japan: estimate = 6.22 × 10^−4^, *z*-value = 2.07, *p* = 0.04 but rvi = 1, table 2). There is also a tendency for the interaction between the gender and the country variables to influence the departure period ([Japan] : [male]; estimate = 5.72 × 10^−4^, *z*-value = 1.88, *p* = 0.059), meaning that men in Japan seem to set off later than men in France. The departure of subsequent pedestrians is not influenced by gender ([male]; estimate = −4.61 × 10^−4^, *z*-value = 1.4, *p* = 0.16), closeness to the kerb (estimate = 3.61 × 10^−5^, *z*-value = 0.59, *p* = 0.55), arrival order (estimate = −2.04 × 10^−6^, *z*-value = 0.075, *p* = 0.94) or age (estimate < |1.07 × 10^−3^|, *z*-value < 0.981, *p* > 0.41).

**Figure 4.**
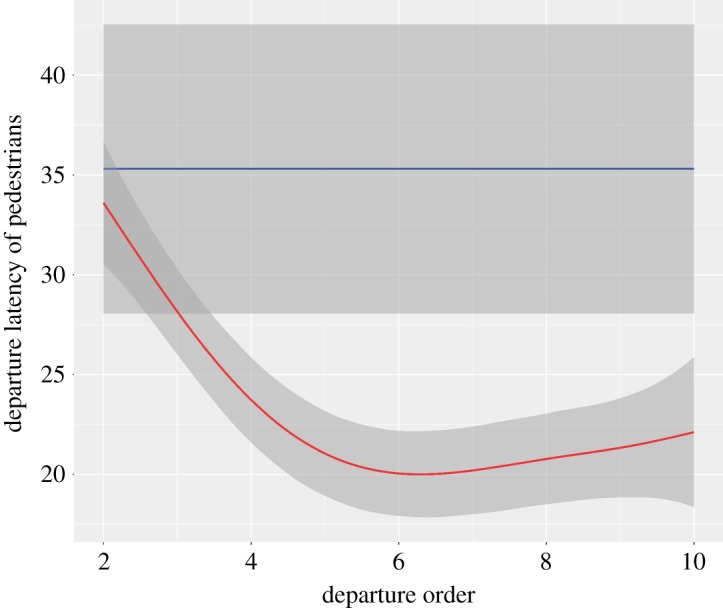
Departure latency of pedestrians according to departure order and country. The departure latency is the time that elapses between the departures of pedestrian *p* and the pedestrian *p* − 1. The red parabolic curve for pedestrians in Japan indicates that pedestrians are influenced by their conspecifics (mimetic process or social information [9,30]), while the uniform blue distribution indicates that pedestrians in France show less inclination to follow social information. Grey shading indicates 0.95 confidence intervals.

**Figure 5.**
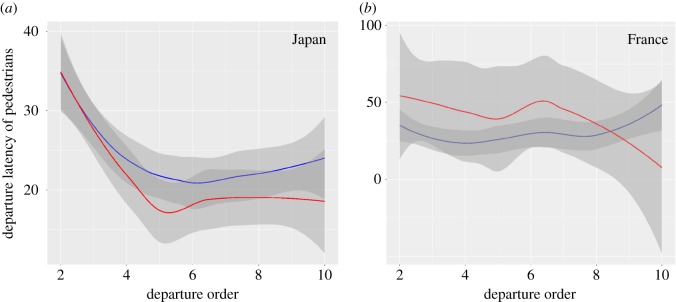
Departure latency of pedestrians according to departure order and gender in Japan (*a*) and in France (*b*). The red and blue curves indicate men and female pedestrians, respectively. Grey shading indicates 0.95 confidence intervals.

The departure latency, meaning the time elapsed between the departures of each pedestrian, is influenced by (i) the number of pedestrians waiting (estimate = 2.43 × 10^−3^, *z*-value = 9.86, *p* < 0.0001) and (ii) the interactions between the country and the departure order variables ([Japan]; estimate = 3.04 × 10^−3^, *z*-value = 3.36, *p* = 0.0008, figure 4), meaning that, depending on the departure order rank, French and Japanese citizens do not behave in the same way. The departure latency is also influenced by the interactions between the gender and the departure order ([male] : [departure order]; estimate = 2.27 × 10^−3^, *z*-value = 2.81, *p* = 0.004, figure 5) and by gender ([male]; estimate = −1.76 × 10^−2^, *z*-value = 2.89, *p* = 0.004). For the last departure order ranks, we can observe that women take more time deciding to start crossing than men, meaning that men take more risks, or that women are more careful, or both. The departure latency is also influenced by the interaction between the country variable and the gender ([Japan] : [male]; estimate = 1.05 × 10^−2^, *z*-value = 2.36, *p* = 0.018), meaning that men and women do not behave in the same way depending on both the country and the departure order rank (figure 5). The closeness to the kerb also tends to play a role (estimate = −2.05 × 10^−3^, *z*-value = 1.8, *p* = 0.07). The country does not influence the departure latency (estimate = −2.64 × 10^−3^, *z*-value = 0.42, *p* = 0.67) even if the variable relative importance is high (rvi = 1, table 2). The time between each pedestrian is not influenced by the arrival order (estimate = 3.02 × 10^−4^, *z*-value = 0.70, *p* = 0.481), the waiting time (estimate = −4.80 × 10^−8^, *z*-value = 0.10, *p* = 0.92) or the time elapsed since the last car passed (estimate = 2.33 × 10^−8^, *z*-value = 0.45, *p* = 0.65), which is quite understandable, because the departure latency is linked with the behaviours of others rather than environmental variables. Likewise, age has no effect on departure latency (estimate < 2.84×10^−1^, *z*-value < 1.14, *p* > 0.25).

### 3.3. Crossing collectively against the red light

**Figure 6.**
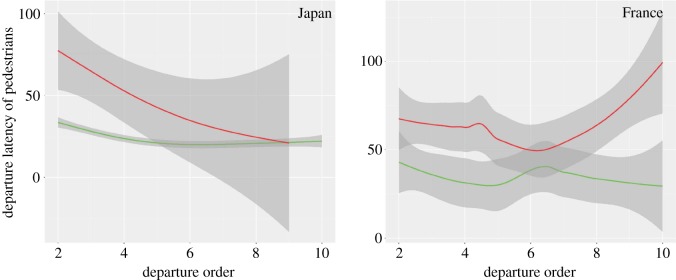
How country and light colour influence the link between the departure order and the departure latency. Green and red curves indicate crossing at the green and red light, respectively. Grey shading indicates 0.95 confidence intervals.

The point of interest here is the behavioural change of pedestrians when someone crosses against the red light. We therefore only recorded interactions between the different variables and this specific light factor. Twice as many pedestrians followed another crossing against the red light in France as in Japan (permutation test for independent samples, *z*-value = 2.6, *p* = 0.009). The departure latency of pedestrians is negatively influenced by the interaction [light colour, red] : [number of waiting pedestrians] (estimate = −2.5 × 10^−4^, *z*-value = 6.25, *p* < 0.0001), meaning that the higher the number of pedestrians waiting at the red light, the slower pedestrians will be to start crossing before it changes. The departure period is therefore affected by the interaction [light colour, red] : [country, Japan] (estimate = −1.69 × 10^−2^, *z*-value = 4.3, *p* < 0.0001), meaning that French and Japanese pedestrians have different behaviours when following other pedestrians who are crossing against the red light (figure 6): Japanese citizens are much faster than French pedestrians when following at the red light, especially for the last ranks. The interaction [departure order] : [light colour, red] negatively influenced the departure latency (estimate = −1.56 × 10^−3^, *z*-value = 2.78, *p* = 0.005), indicating that pedestrians are slower to start crossing when they follow someone at the red light, possibly because they check the risk before stepping off the kerb. Finally, the departure latency is positively influenced by the interaction [light colour, red] : [waiting time] (estimate = 1.6 × 10^−6^, *z*-value = 2.3, *p* = 0.017), showing that pedestrians are less hesitant about following another pedestrian across the road at the red light when they have been waiting for a long period of time. Departure latency is not affected by the interactions [light colour, red] : [gender, male] (estimate = −1.7 × 10^−3^, *z*-value = 0.7, *p* = 0.48), [light colour, red] : [closeness to the kerb] (estimate = 1.89 × 10^−3^, *z*-value = 1.07, *p* = 0.28) or [light colour, red] : [last car time] (estimate = −2.34 × 10^−8^, *z*-value = 0.34, *p* = 0.73). Even if the relative variable importance equals 0.99, age does not influence departure latency when in interaction with the light colour (estimate < 4.6 × 10^10^, *z*-value = 0.09, *p* = 0.92).

## 4. Discussion

Our culture influences the way we live and how we behave. This study seeks to assess how pedestrians use social information to cross the road in cities of two countries with different social norms. The results reveal that road-crossing behaviour is influenced not only by our country of residence, but also by many other environmental, personal or social factors. This confirms previous studies and provides new perspectives for pedestrian safety applications.

### 4.1. Influence of road environment variables

The number of lanes has an effect on the probability of a pedestrian crossing against the red light, with the lowest incidence of rule-breaking recorded at locations with a high number of lanes. This is logical, as there were no raised medians on the sites we studied, except one in the middle of the 2 × 3 lanes near the Nagoya train station. The number of lanes increased the acceptable distance of the next car for pedestrians, but they considered the safe distance to be shorter when in the presence of a pedestrian refuge island [29,31]. A previous study [36] showed that refuge islands increase the crossing rate by 5.4%, but still concluded that refuge islands make it significantly safer for pedestrians to cross the road. This kind of infrastructure may therefore be advantageous in so far as it prevents accidents and decreases the number of pedestrian accidents with cars by half, but it could also encourage pedestrians to cross against the light and therefore increase this illegal behaviour.

Waiting time before crossing was also shown to have an influence on crossing behaviour. The longer individuals waited at the pedestrian crossing, the more likely they were to leave first and cross the road quickly. A previous study [36] showed that for every extra second spent waiting at the red light, the rate of rule-breaking increased by 0.017%. We also found that pedestrians were less hesitant to follow someone crossing against the red light when they had been waiting at the crossing for a long time. This point is important, as these results indicate that pedestrians may have a maximum waiting time, above which they will decide to cross against the red light [37]. This study does not assess a specific threshold in this study, but this issue should be examined in further studies. Human beings have often shown evidence of using a decision threshold process [9,17], and the evaluation of the ideal waiting time would decrease the occurrence of rule-breaking and the number of pedestrian accidents.

### 4.2. Influence of personal characteristics

Our study confirms the results of previous studies and provides evidence that gender affects crossing behaviours. Men crossed against the red light more often than women. Men were also the first to cross the road when the light turned green. However, male pedestrians in Japan showed a tendency to cross later or slower than French counterparts. Our results confirm that women seem to be more careful than men [8,19] or that men showed more risky behaviours as a sexual strategy, as described in the sociobiological theory of male competitiveness [21]. A previous study [38] explained that women could also display higher conformity to group pressure and more compliance to legal prohibition. However, the study finally showed that women were no more compliant than men, but were apparently more conformist. This may be confirmed by the difference between men and women for departure latency according to the departure order in our results (figure 5), where women showed a stronger mimetic behaviour than men. This mimetic behaviour is an expression of conformism when we adapt our behaviour to that of the majority. Conformism is well described in humans [39,40] but has also been observed in non-human primates [41], where the more philopatric sex (i.e. the one remaining in the native group) as well as the migrating sex might decide to follow the majority to ensure their integration within the group: the same conclusion might be reached in humans. It is interesting to note that, in other animal species, individuals of different age, social status or gender also cross the road in different ways [42–45]. In meerkats (*Suricata suricatta*), the dominant female is the last one to cross the road and crossing at the last position is the less risky behaviour [44]. In chimpanzees (*Pan troglodytes*), dominant individuals act cooperatively to maximize group protection during road crossing, with 20% of crossing individuals paying attention to group members by checking on them or waiting for them while crossing. Ninety per cent of the studied chimpanzees looked right and left before crossing [42,43]. More detailed studies of the origins of road-crossing behaviours in animals could widen our understanding of behavioural differences between human beings.

Individuals aged 20–30 years showed a greater tendency to cross against the red light than other pedestrians. This was not the case for very young or old people. Nor did we find any effect of age on the order of departure or the departure period. Usually, the higher rate of rule-breaking in adolescents or young adults is explained not only by their willingness to participate in risky and competitive interactions as previously described [21], but also by the peer pressure of same-age conspecifics for both genders [46]. Young adults want not only to affirm their position in society, but also need to obtain the acceptance of others, and particularly people belonging to the same age category. Peers do not necessarily have to be present to influence pedestrian behaviours; previous comments might well influence the subsequent actions and decisions of the recipients. Pfeffer & Hunter concluded in their study [46] that we should use this peer influence as a source of learning and a means to prevent injury risk.

### 4.3. Influence of social variables

The number of illegal crossings (crossing against the red light) is 41.9% in France and only 2.1% in Japan. This difference between France and Japan was already noted in our first study of pedestrians crossing the road alone [17]. However, the same study revealed that the rate of illegal crossings when pedestrians were not located close to other people was 67% in France and 6.9% in Japan. This means that the number of pedestrians crossing against the red light is much lower when other pedestrians are present (67% versus 41.9%, or −37% in France, and 6.9% versus 2.1%, or −69.6% in Japan), indicating a strong social influence. Similarly, this study shows that pedestrians displayed a lower number of illegal crossings when they are accompanied by familiar individuals. We can explain this result by a simple mimetic effect, where the probability of not crossing increases with the number of waiting pedestrians [30,37]. Individuals might also interpret this social information of waiting at the pedestrian crossing as a higher risk of crossing against the red light, but this explanation is highly unlikely. A feasible explanation would be that pedestrians conform to group pressure [38]: individuals tend to adopt the behaviour of others in order to retain their social credibility, and this concept is particularly true in the collectivist societies of countries like Japan [27]. The Japanese are more respectful of the rules than French citizens but are particularly aware of the opinion of conspecifics [22,47]. Indeed, results show a greater decrease in the rate of rule-breaking when other people are present in Japan compared with France. People who would cross against the red light when alone do not cross when other pedestrians are present and waiting because they are more afraid of being criticized than they are of being fined.

The main result of our study is the great impact of the country of residence on the road-crossing behaviours of pedestrians. The differences between the two countries are probably a reflection of differences in culture. In order to confirm this, it would be interesting to not only study different sites in other countries than France and Japan, but also repeat this study at other sites in the same countries to investigate possible geographical variation in road-crossing behaviour. This will make it possible to better identify measurable cultural behaviours to adapt road design to the populations using them. The first pedestrian to cross at the green light steps off the kerb sooner in France than in Japan. This confirms that although the more individualistic behaviour in Western countries makes people more autonomous, it also entails more risky behaviour [17]. Indeed, while the sites are comparable between the two countries, pedestrians in France leave sooner than their Japanese counterparts, thus increasing their risk of injury as they take less time to check for their safety. This might be explained by the fact that the Japanese are more risk averse than the French, or may indicate that the Japanese observe the behaviours of other pedestrians before crossing. The second hypothesis reflects the concept of conformism but is a less plausible explanation than risk aversion.

The different extents to which mimetism (likelihood to follow) underlies the probability of crossing in Strasbourg and Nagoya is also illustrated by the influence of the number of waiting individuals on the departure period. This mimetic effect or so-called *information cascade* is different in Japanese and French pedestrians. Although the first pedestrian is quicker to decide to cross when the light turns green in France compared with his or her counterpart in Japan, we observed that the subsequent decisions of other pedestrians to cross occurred much faster in Japan than in France. This strong mimetic effect is shown by the parabolic curve in figure 4: Japanese pedestrians follow others according to the social information they obtain, and apply highly conformist behaviour. Indeed, the information cascade is the very basis of conformism [48]. Contrarily, French pedestrians do not show any evidence of this strong mimetic effect. This means that even when the light is green and pedestrians are crossing, French people have less confidence in the social information (meaning that they check the light colour) and/or do not show the same strong conformism in crossing as Japanese pedestrians.

Japanese pedestrians are, however, shown to follow social information less when crossing against the red light. They were half as likely to follow a pedestrian crossing against the red light as French people and when they did so they started to move faster than their French counterparts, indicating that they were aware of the risk they were taking. This means that pedestrians make different use of social information according to the situation in hand. We can at least conclude from this study that Japanese people show the use of social information to improve their social integration (‘I follow others when the light is green’) and decrease their risk of injury (‘I do not follow others when the light is red’). We cannot reach the same conclusion for French pedestrians, who prefer to rely on their own information. Theoretically, pedestrians following social information should be at higher risk than those using their own information owing to uncertainty or incorrect decisions that may cause an accident. In this way, Faria *et al.* [8] concluded that social information might be disadvantageous in some circumstances as the distance between the crossing point and an oncoming car for a second or a third pedestrian is much shorter than when the first pedestrian decided to cross the road at the red light, thus increasing the risk of injury. However, surprisingly, there is little difference between the number of road fatalities per 100 000 inhabitants per year in France and Japan (respectively, 5.5 and 4.7, approx. 30% of whom were pedestrians) [49]. This raises the question of the utility of social information use for road safety application, because there is no difference between the use of personal and social information, whatever the light colour. This result brings us back to the spontaneous emergence of conventions [28]: social information use and conformism in Japanese society do not provide any advantage to Japanese people other than maintaining a certain credibility in it [50].

## 5. Conclusion

We found that the light colour is the main factor influencing the crossing behaviours of pedestrians, closely followed by the departure order and the country of pedestrians. The number of waiting pedestrians and their gender also have strong effects on the way they will cross.

To enhance pedestrian safety, local authorities are seeking a combination of appropriate interventions that are often referred to as the three Es—engineering, enforcement and education. However, we can conclude that, with the exception of the light colour, the majority of factors affecting our behaviours are not environmental but are mainly linked to personal, social or cultural human characteristics. Although better road design or urban planning may help to globally decrease the risk of injury on the road, a decrease in the number of accidents could be attained through a better understanding of how human beings behave and the identification of mechanisms underlying behavioural differences between individuals of different genders, ages and cultures, thus providing a better basis for prevention and education. For instance, much remains to be learned about the influence of peers, which could play an important role in preventive measures [46]. Specific ringing signals could also be designed and set up in order to prevent further pedestrians from following a first individual crossing against the red light. Current technologies can provide the means to identify many different behaviours [9], and their use in pedestrian crossings could play a role in decreasing risk and preventing accidents.

## Supplementary Material

Supplementary information file for “Cultural influence of social information use in pedestrian road-crossing behaviours”Table S1 : Statistical values (Z-value and P-value) for each GzLM. * : the light color condition is not anymore significant since the variable was tested in interaction with all other variables. Figure S1: Picture of the site “Train Station”, Strasbourg, France Figure S2: picture of the site “Pont des Corbeaux”, Strasbourg, France Figure S3: picture of the site “Place Broglie”, Strasbourg, France Figure S4: picture of the site “Train Station”, Nagoya, Japan Figure S5: picture of the site “Maruei”, Nagoya, Japan Figure S6: picture of the site “Excelco”, Nagoya, JapanFigure S7: Figure of the site “Osu-Kannon”, Nagoya, JapanFigure S8: Graphs showing the clustering results of the hierarchal clustering analysis following a Principal component analysis. a.) Factor map showing the distribution of data according to two PCA dimensions and the clusters in different colours. b.) Variables factor map showing the contributions and correlations of the different variables to the dimensions. c.) Dendogram showing how the analysis clustered data. Table S2: Variance for each dimension given by the Principal component analysisTable S3: Correlations of the variables to the dimensions given by the Principal Component AnalysisTable S4: Contributions of the variables to the dimensions given by the Principal Component AnalysisTable S5: Correlations (for quantitative variables) or regression coefficients (for qualitative ones) between varaibles and dimensions as well as ones p-values indicating how variables participate to the variance of dimensions.

## Data accessibility

Data are available from the Dryad Digital Repository: http://dx.doi.org/10.5061/dryad.tc365 [51].
