# Supplementary material for: Cultural influence of social information use in pedestrian road-crossing behaviours
Source: R Soc Open Sci. 2017 Feb 15;4(2):160739. doi: 10.1098/rsos.160739 (PMC5367276; doi:10.1098/rsos.160739)
Supplement: Supplementary information file for “Cultural influence of social information use in pedestrian road-crossing behaviours”Table S1 : Statistical values (Z-value and P-value) for each GzLM. * : the light color condition is not anymore significant since the variable was tested in interaction with all ot [file rsos160739supp1.docx]

Supplementary information file

**Cultural influence of social information use in pedestrian road-crossing behaviours**

Marie Pelé^1^, Caroline Bellut^2,3^, Elise Debergue^2,3^, Charlotte Gauvin^2,3^, Anne Jeanneret^2,3^, Thibault Leclere^2,3^, Lucie Nicolas^2,3^, Florence Pontier^2,3^, Diorne Zausa^2,3^, Cédric Sueur^2,3,4^

^1^ Ethobiosciences, Research and Consultancy Agency in Animal Wellbeing and Behaviour, Strasbourg, France

^2^ Centre National de la Recherche Scientifique, Département Ecologie, Physiologie et Ethologie, Strasbourg, France

^3^ Université de Strasbourg, Institut Pluridisciplinaire Hubert Curien, Strasbourg, France

^4^ Primate Research Institute, Kyoto University, Inuyama, Japan


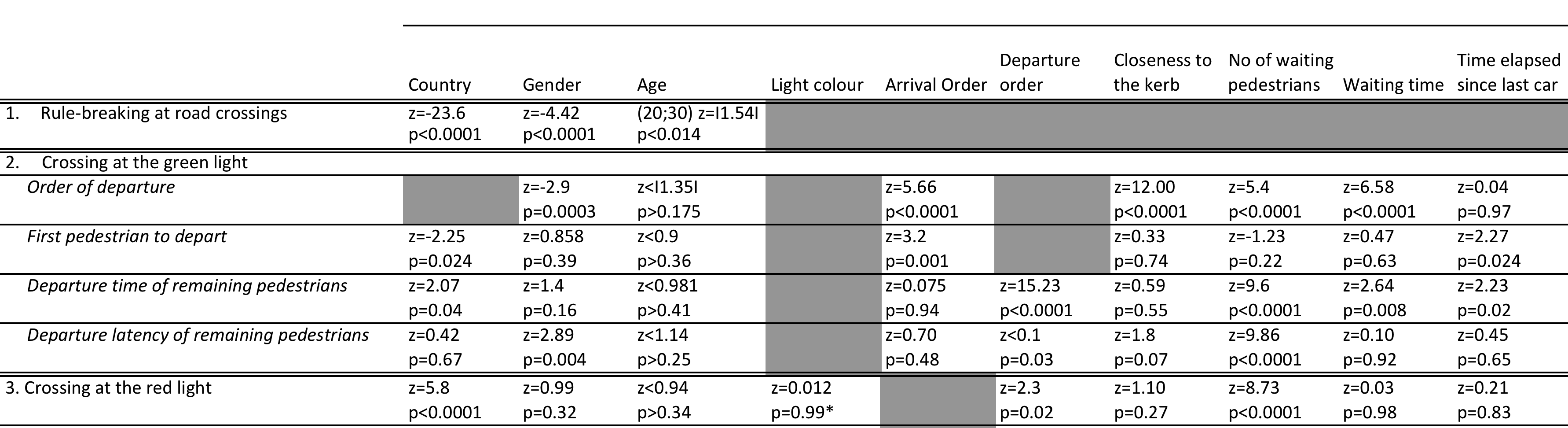


Table S1 : Statistical values (Z-value and P-value) for each GzLM. * : the light color condition is not anymore significant since the variable was tested in interaction with all other variables.


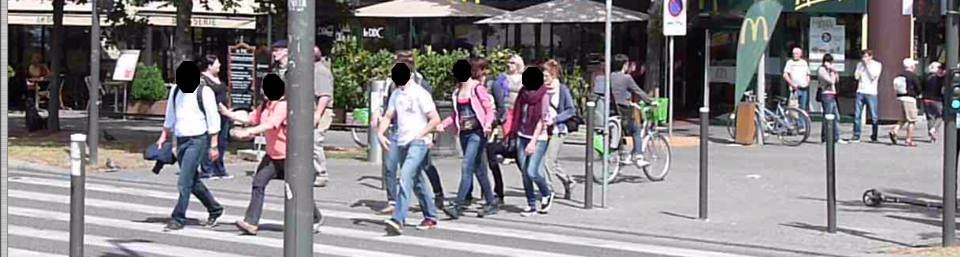


Figure S1: Picture of the site “Train Station”, Strasbourg, France


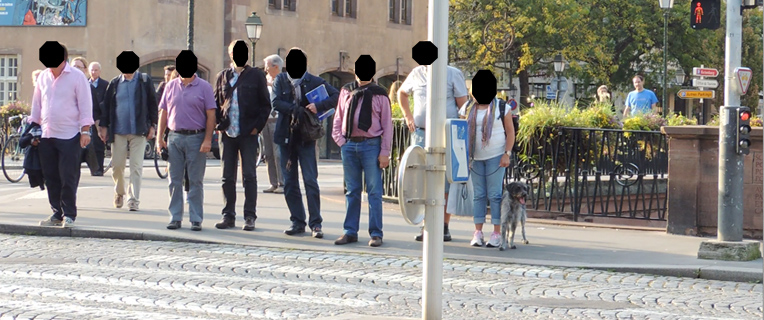


Figure S2: picture of the site “Pont des Corbeaux”, Strasbourg, France


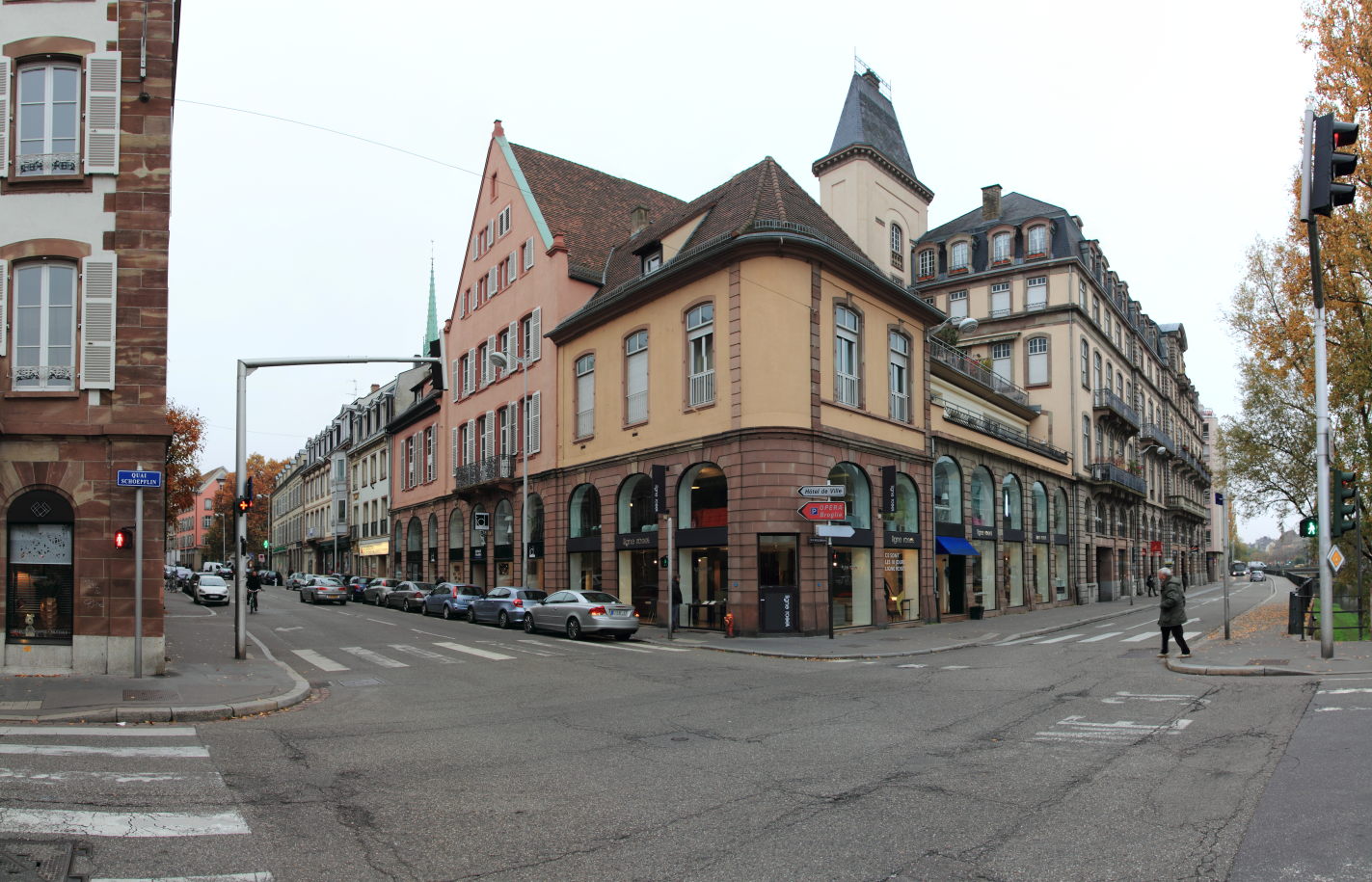


Figure S3: picture of the site “Place Broglie”, Strasbourg, France


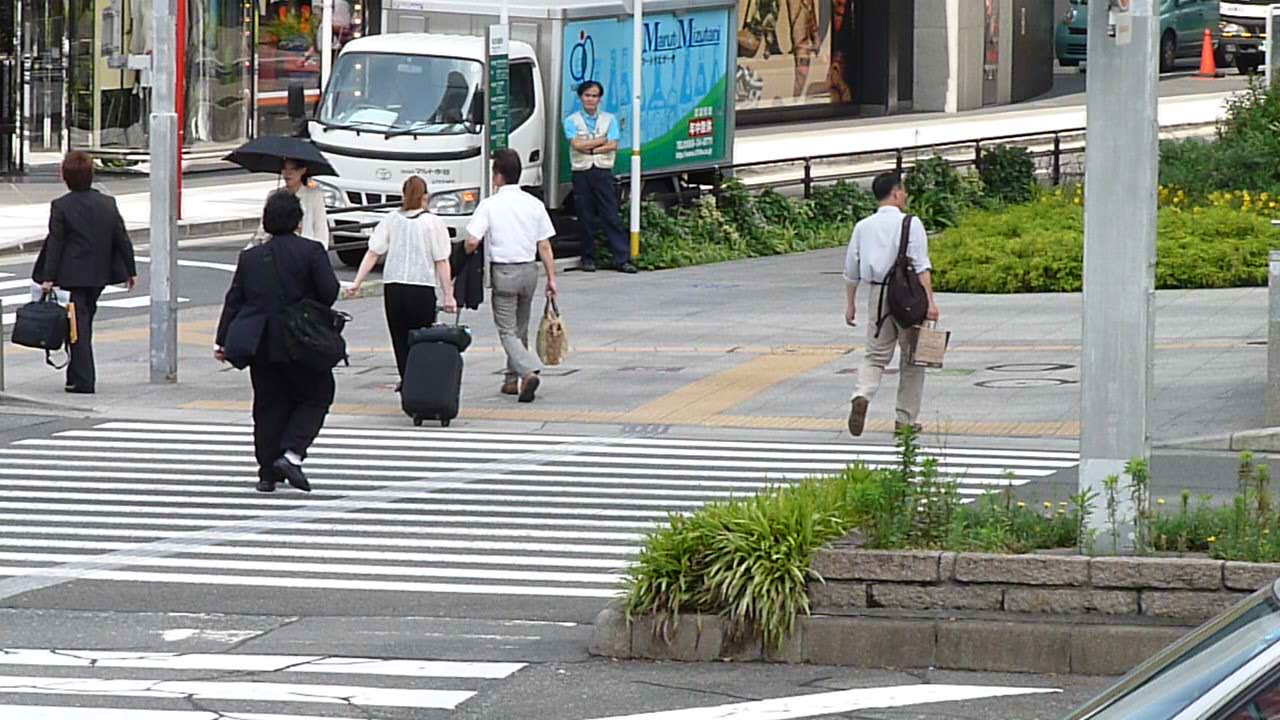


Figure S4: picture of the site “Train Station”, Nagoya, Japan


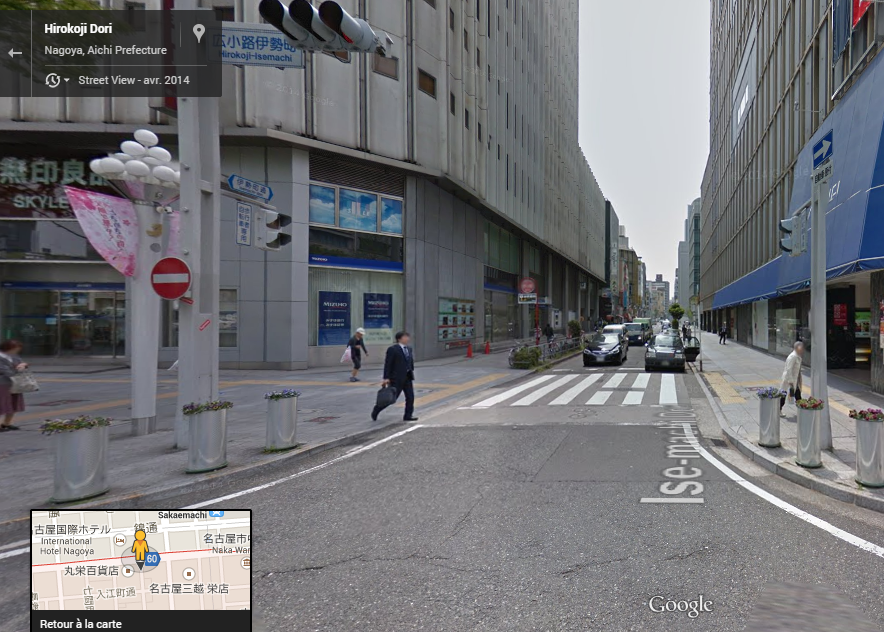


Figure S5: picture of the site “Maruei”, Nagoya, Japan


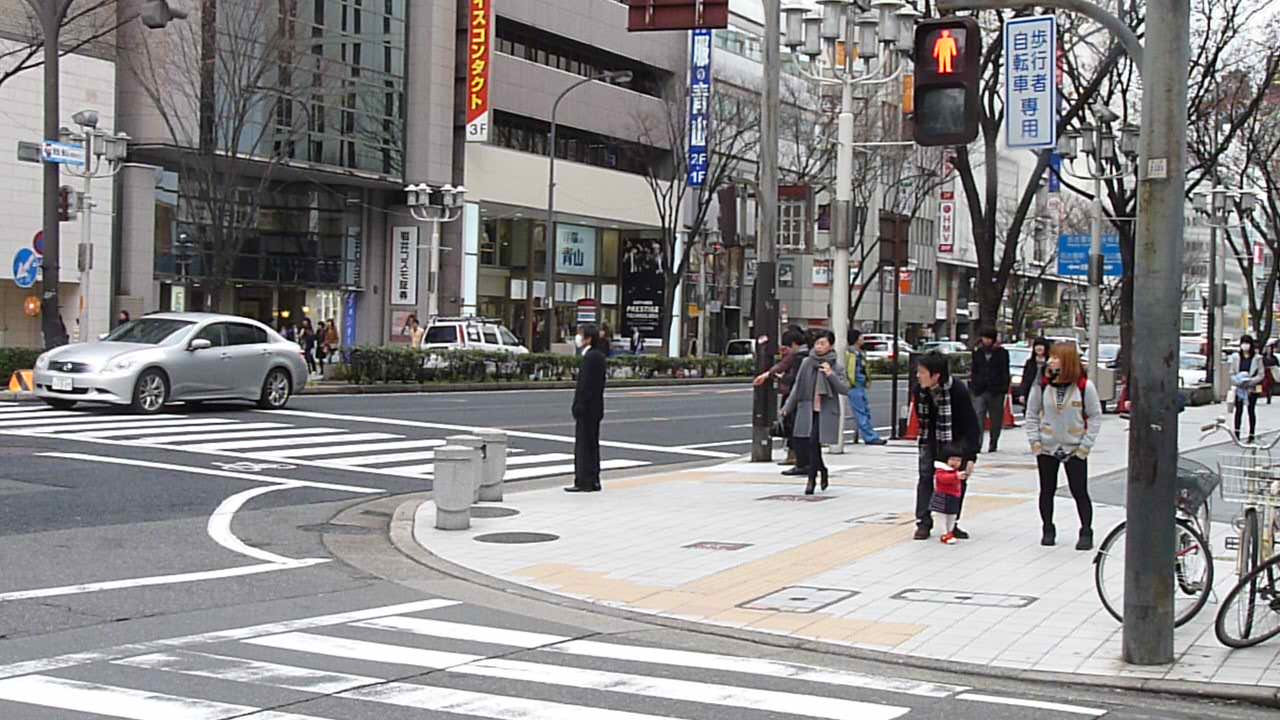


Figure S6: picture of the site “Excelco”, Nagoya, Japan


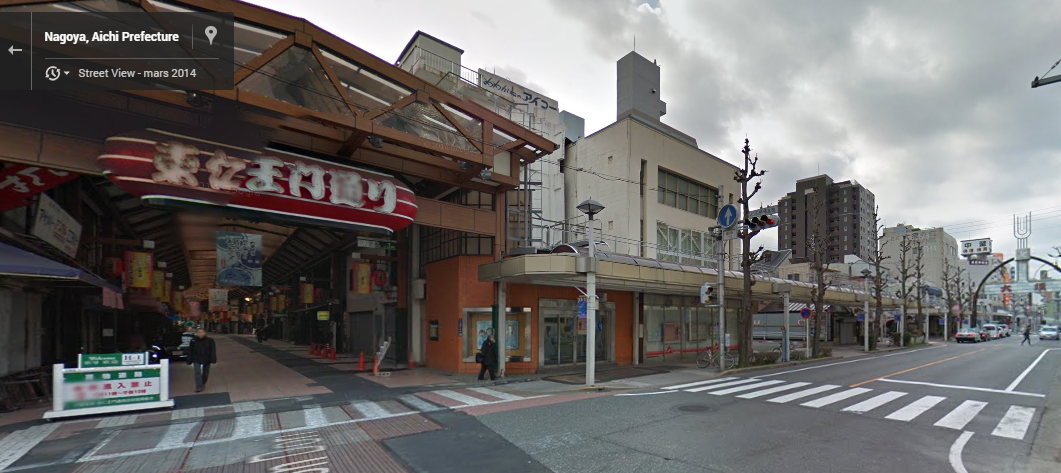
Figure S7: Figure of the site “Osu-Kannon”, Nagoya, Japan


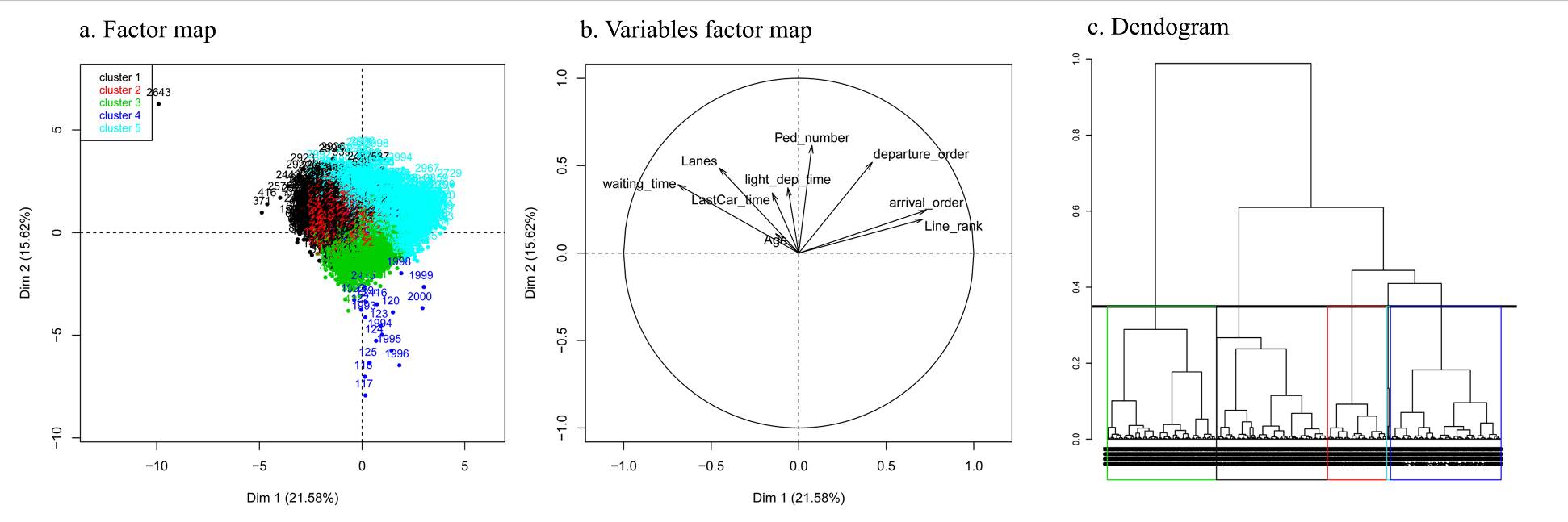


Figure S8: Graphs showing the clustering results of the hierarchal clustering analysis following a Principal component analysis. a.) Factor map showing the distribution of data according to two PCA dimensions and the clusters in different colours. b.) Variables factor map showing the contributions and correlations of the different variables to the dimensions. c.) Dendogram showing how the analysis clustered data.

Table S2: Variance for each dimension given by the Principal component analysis

| Eigenvalues | Dim.1 | Dim.2 | Dim.3 | Dim.4 | Dim.5 | Dim.6 | Dim.7 | Dim.8 | Dim.9 |
| --- | --- | --- | --- | --- | --- | --- | --- | --- | --- |
| Variance | 1.942 | 1.406 | 1.124 | 1.019 | 0.930 | 0.886 | 0.822 | 0.561 | 0.309 |
| % of var. | 21.583 | 15.622 | 12.492 | 11.326 | 10.339 | 9.843 | 9.131 | 6.230 | 3.434 |
| Cumulative % of var. | 21.583 | 37.205 | 49.697 | 61.023 | 71.362 | 81.205 | 90.337 | 96.566 | 100.000 |

Table S3: Correlations of the variables to the dimensions given by the Principal Component Analysis

| $cor | Dim.1 | Dim.2 | Dim.3 | Dim.4 | Dim.5 |
| --- | --- | --- | --- | --- | --- |
| light_dep_time | -0.06193652 | 0.3736864 | -0.07337054 | 0.70114972 | 0.50233319 |
| departure_order | 0.42002977 | 0.5191889 | -0.34413304 | -0.26006262 | 0.09499513 |
| arrival_order | 0.72889949 | 0.2449524 | 0.41903808 | 0.05132838 | -0.08733171 |
| waiting_time | -0.68942404 | 0.3908750 | -0.40901429 | -0.14348873 | 0.07680724 |
| Age | -0.13191345 | 0.1103439 | 0.67242475 | -0.10000063 | 0.36410134 |
| Kerb closeness | 0.70943926 | 0.1933193 | -0.30738005 | 0.09415925 | 0.14880158 |
| LastCar_time | -0.15148232 | 0.3442142 | 0.09303431 | 0.52496909 | -0.67457601 |
| Ped_number | 0.07554985 | 0.6158399 | 0.11616800 | -0.36505545 | -0.19806886 |
| Lanes | -0.45417526 | 0.4865384 | 0.29797572 | -0.09563428 | 0.08132829 |

Table S4: Contributions of the variables to the dimensions given by the Principal Component Analysis

| $contrib | Dim.1 | Dim.2 | Dim.3 | Dim.4 | Dim.5 |
| --- | --- | --- | --- | --- | --- |
| light_dep_time | 0.1974847 | 9.931875 | 0.4788190 | 48.2284914 | 27.1186377 |
| departure_order | 9.0823863 | 19.172004 | 10.5336923 | 6.6349554 | 0.9698102 |
| arrival_order | 27.3511206 | 4.267564 | 15.6183399 | 0.2584623 | 0.8196492 |
| waiting_time | 24.4688003 | 10.866571 | 14.8800659 | 2.0198436 | 0.6339988 |
| Age | 0.8958143 | 0.865991 | 40.2175190 | 0.9810420 | 14.2471718 |
| Line_rank | 25.9101698 | 2.658074 | 8.4038688 | 0.8697774 | 2.3795740 |
| LastCar_time | 1.1813095 | 8.427023 | 0.7698643 | 27.0364469 | 48.9041709 |
| Ped_number | 0.2938374 | 26.974434 | 1.2003307 | 13.0737394 | 4.2161545 |
| Lanes | 10.6190771 | 16.836464 | 7.8975001 | 0.8972415 | 0.7108328 |

Table S5: Correlations (for quantitative variables) or regression coefficients (for qualitative ones) between varaibles and dimensions as well as ones p-values indicating how variables participate to the variance of dimensions.

|  | Dim 1 | | Dim 2 | | Dim 3 | | Dim 4 | | Dim 5 | |
| --- | --- | --- | --- | --- | --- | --- | --- | --- | --- | --- |
|  | correlation/R2 | p.value | correlation/R2 | p.value | correlation/R2 | p.value | correlation/R2 | p.value | correlation/R2 | p.value |
| arrival_order | 0.72889949 | 0.000000e+00 | 0.6158399 | 3.660236e-315 | 0.67242475 | 0.000000e+00 | 0.70114972 | 0.000000e+00 | 0.50233319 | 4.917238e-193 |
| Line_rank | 0.70943926 | 0.000000e+00 | 0.5191889 | 2.472437e-208 | 0.41903808 | 7.429655e-129 | 0.52496909 | 8.820686e-214 | 0.36410134 | 2.008881e-95 |
| departure_order | 0.42002977 | 1.612463e-129 | 0.4865384 | 1.856004e-179 | 0.29797572 | 4.900449e-63 | 0.09415925 | 2.143620e-07 | 0.14880158 | 1.971233e-16 |
| Ped_number | 0.07554985 | 3.209149e-05 | 0.3908750 | 6.378212e-111 | 0.11616800 | 1.492059e-10 | 0.05132838 | 4.760314e-03 | 0.09499513 | 1.670690e-07 |
| light_dep_time | -0.06193652 | 6.562213e-04 | 0.3736864 | 8.350883e-101 | 0.09303431 | 2.988098e-07 | -0.09563428 | 1.378808e-07 | 0.08132829 | 7.564760e-06 |
| Age | -0.13191345 | 3.296837e-13 | 0.3442142 | 7.845004e-85 | -0.07337054 | 5.395021e-05 | -0.10000063 | 3.593286e-08 | 0.07680724 | 2.363013e-05 |
| LastCar_time | -0.15148232 | 5.584031e-17 | 0.2449524 | 1.509991e-42 | -0.30738005 | 3.676859e-67 | -0.14348873 | 2.243955e-15 | -0.08733171 | 1.517936e-06 |
| Lanes | -0.45417526 | 9.026933e-154 | 0.1933193 | 7.686688e-27 | -0.34413304 | 8.635056e-85 | -0.26006262 | 6.398841e-48 | -0.19806886 | 4.049006e-28 |
| waiting_time | -0.68942404 | 0.000000e+00 | 0.1103439 | 1.179672e-09 | -0.40901429 | 2.838671e-122 | -0.36505545 | 5.963254e-96 | -0.67457601 | 0.000000e+00 |
| Sex | 0.009162216 | 1.343846e-07 | / | / | 0.02315657 | 4.020048e-17 | / | / | / | / |
| light_color | / | / | 0.08031330 | 6.107736e-57 | 0.02700984 | 9.524683e-20 | / | / | 0.04975108 | 2.158211e-35 |
| Country | / | / | 0.06931731 | 4.115005e-49 | 0.02315657 | 4.020048e-17 | 0.01025002 | 2.425634e-08 | 0.04803431 | 3.355213e-34 |
